# Frequency of microbial isolates and pattern of antimicrobial resistance in patients with hematological malignancies: a cross-sectional study from Palestine

**DOI:** 10.1186/s12879-022-07114-x

**Published:** 2022-02-10

**Authors:** Genan Arman, Marwa Zeyad, Beesan Qindah, Adham Abu Taha, Riad Amer, Shatha Abutaha, Amer A. Koni, Sa’ed H. Zyoud

**Affiliations:** 1 Department of Medicine, College of Medicine and Health Sciences, An-Najah National University, Nablus, 44839 Palestine; 2 Department of Biomedical Sciences, Faculty of Medicine and Health Sciences, An-Najah National University, Nablus, 44839 Palestine; 3 Department of Pathology, An-Najah National University Hospital, Nablus, 44839 Palestine; 4 Department of Hematology and Oncology, An-Najah National University Hospital, Nablus, 44839 Palestine; 5 Department of Clinical and Community Pharmacy, College of Medicine and Health Sciences, An-Najah National University, Nablus, 44839 Palestine; 6 Division of Clinical Pharmacy, Department of Hematology and Oncology, An-Najah National University Hospital, Nablus, 44839 Palestine; 7 Poison Control and Drug Information Center (PCDIC), College of Medicine and Health Sciences, An-Najah National University, Nablus, 44839 Palestine; 8 Clinical Research Center, An-Najah National University Hospital, Nablus, 44839 Palestine; 9 Department of Pharmacy, College of Medicine and Health Sciences, An-Najah National University, Nablus, 44839 Palestine

**Keywords:** Bacterial isolates, Antimicrobial resistance, Hematologic malignancies, Palestine

## Abstract

**Background:**

Infections are the main cause of death in patients with hematologic malignancies. This study aims to determine the microbial profile of infections in patients with hematologic malignancies and to determine the antimicrobial resistance patterns for these pathogens.

**Methods:**

A retrospective descriptive cross-sectional study was conducted from January 2018 to December 2019 at a large hematological center in Palestine. The medical data of hematologic malignancy patients with positive cultures were collected from the hematology/oncology department using the hospital information system, and data regarding the microbial isolates and their antimicrobial resistance were collected from the microbiology laboratory.

**Results:**

A total of 144 isolates were identified from different types of specimens, mostly blood samples. Of all isolates, 66 (45.8%) were gram-negative bacteria (GNB), 57 (39.6%) were gram-positive bacteria (GPB), and 21 (14.6%) were fungal isolates. The GNB that were most frequently isolated were *Pseudomonas aeruginosa* (27, 40.9%), followed by *Escherichia coli* (*E. coli*) (20, 30.3%). Fourteen isolates (24.6%) of GPB were *Staphylococcus epidermidis* followed by *Enterococcus faecium* (10, 17.5%) and *Staphylococcus hemolyticus* (10, 17.5%)*.* The most frequent fungal pathogens were *Candida* species (20, 95.2%). GNB were found to be resistant to most antibiotics, mainly ampicillin (79.3%). *Pseudomonas aeruginosa* exhibited high resistance to ciprofloxacin (60%) and imipenem (59.3%). Among GPB, high resistance rates to oxacillin (91.1%) and amikacin (88.8%) were found. All isolated strains of *Staphylococcus epidermidis* were resistant to cephalosporins and oxacillin. Approximately half of the GNB isolates (34, 51.5%) were multi-drug resistant organisms (MDRO), and 16.7% (11 isolates) were difficult-to-treat resistance (DTR). Furthermore, 68.4% (39 isolates) of GPB were MDRO. The proportion of staphylococci (CoNS and *S. aureus*) resistant to oxacillin was 91.7%, while 88.6% of enterococci were resistant to vancomycin.

**Conclusions:**

The findings of this study confirm the predominant microorganisms seen in patients with hematologic malignancies, and show a high percentage of antibiotic resistance. Policies regarding antibiotic use and proper infection control measures are needed to avert the ever-growing danger of antimicrobial resistance. This may be achieved by developing antibiotic stewardship programs and local guidelines based on the hospital's antibiogram.

## Background

In recent decades, major developments have been made in the care of cancer patients that have significantly improved patient survival. However, despite these developments, patients with hematologic malignancies remain at an extraordinarily high risk of infections. This is the result of a complex interaction between basic immunodeficiency and therapeutic practices such as surgery, radiation, and chemotherapy [1–4].

Previous studies have reported that the prevalence of bacterial bloodstream infections among patients with hematologic malignancies ranges from 11 to 38%, and the rough mortality rate reaches up to 40% [5–7]. Furthermore, studies have shown gram-negative bacteria (GNB) to be the most frequently isolated organisms during the 1960s and 1970s [8]. However, over the following two decades, the proportion of gram-positive bacteria (GPB) has increased.

A study in India showed that *E. coli* was the most common isolated organism, followed by coagulase-negative staphylococci (CoNS) [9]. Another study showed that GNB were the most common isolated organisms, and the best empirical treatment for them was non-carbapenem-based anti-pseudomonal antibiotics [5]. However, another study conducted in 2018 in Ethiopia showed a change in prevalence from GNB to GPB, mainly as a result of increased use of urinary catheters, and multi-drug resistance was detected in 46.3% of bacterial isolates [10].

In Palestine, cancer is the third leading cause of death after heart disease and cerebrovascular disease, accounting for 10.3% of total deaths [11]. However, Palestinian cancer services started to spring up in the early 2000s, and it took until 2008 to organize cancer care at Augusta Victoria Hospital in East Jerusalem [12]. In the Gaza Strip, a study showed a high prevalence of resistance to amoxicillin (73.2%) in isolates of *Staphylococcus aureus*, and high resistance to penicillin (40.4%) in *Streptococcus pneumonia* [13]. However, no published reports have shown the epidemiology and antimicrobial resistance patterns of potential etiological agents among patients with hematologic malignancies in Palestine.

Antibiotic resistance is a growing concern in global health [14–20]. Overuse and constant consumption of antibiotics, due to lack of control programs in hospitals and over-the-counter antibiotics, lead to multi-drug resistant pathogens. These then lead to increased mortality, length of hospitalizations, and health care costs [21, 22].

This study provides information on the spectrum of microbial isolates and their antimicrobial resistance patterns in patients with hematologic cancer at An-Najah National University Hospital. This study is the first to evaluate bacterial and fungal resistance patterns among hematological malignancies in Palestine. This information will help decrease morbidity and mortality by helping to establish empirical treatment guidelines and antibiotic stewardship programs. These will reduce antibiotic overuse and, subsequently, decrease hospitalizations. This study also highlights the immense effect and burden of multi-drug resistant organisms.

## Methods

### Study design

A retrospective cross-sectional study was conducted to determine the frequency of microbial isolates and patterns of antimicrobial resistance in patients with hematologic malignancies.

### Study setting

Data were collected from the medical laboratory department and the hematology department of An-Najah National University Hospital. The hospital has a bed capacity of 169, with approximately 40 beds for adult hematology/oncology patients [23].

### Study population

All patients with hematologic malignancies who had a positive culture from January 2018 to December 2019 at An-Najah National University Hospital.

### Data collection

Demographic and medical data were obtained from the hospital information system. Information on the sources of specimens, types of microorganisms, and antibiotic susceptibility was collected from the microbiology laboratory. Importantly, cefepime is available in the hospital, but its use is very restricted because it requires a special request from an infectious disease specialist. Furthermore, ceftolozane / tazobactam is not available in Palestine.

### Inclusion criteria

All patients with hematologic malignancies who had positive cultures during the study period at An-Najah National University Hospital were included.

### Exclusion criteria

Patients who did not have a positive culture and those who had solid tumors were excluded.

### Ethical considerations

The proposal was reviewed and accepted on September 22, 2019, with the permission of An-Najah National University Hospital. The approval of the Institutional Review Board (IRB) Committee of An-Najah National University was obtained on July 24, 2019 (Archived number: AN4June2019).

### Statistical analysis

Data were entered and analyzed using version 21 of the Statistical Package for Social Sciences (SPSS) program. For continuous variables, data were expressed as means ± standard deviation (SD) and as frequencies and percentages for categorical variables.

## Results

### Demographic and clinical characteristics of the study population

A total of 144 cancer patients were included in the study. Of these, 77 (53.5%) were women and 67 (46.5%) were men. The mean age ± SD of the study participants was 40.8 ± 16.6 years, ranging from 17 to 84 years old. The most common hematologic malignancy was acute lymphoid leukemia (ALL) (36, 25%), followed by Hodgkin’s lymphoma (33, 22.9%), acute myeloid leukemia (30, 20.8%), multiple myeloma (22, 15.3), non-Hodgkin lymphoma (16, 11.1%), chronic lymphoid leukemia (4, 2.8%), Waldenstrom macroglobulinemia (2, 1.4%) and Langerhans cell histiocytosis (1, 0.7%). Most of these patients were actively on the chemotherapy protocol (83, 57.6%) or had had bone marrow transplantation (35, 24.3%), while others had finished their treatment (17, 11.8%) or had not received any cancer treatment at the time their samples were collected (9, 6.3%). It should be noted that most of the patients were febrile (91, 63.2%) and a portion had died at the end of the study (12, 8.3%); (Table 1).

**Table 1:** Demographic and clinical characteristics of cancer patients with positive cultures

Variable		n (%)
Age(years), mean ± SD		40.8 ± 16.6
Sex	Male	67 (46.5)
	Female	77 (53.5)
Type of cancer	ALL	36 (25)
	Hodgkin’s lymphoma	33 (22.9)
	AML	30 (20.8)
	MM	22 (15.3)
	Non-Hodgkin's lymphoma	16 (11.1)
	CLL	4 (2.8)
	Waldenstrom macroglobulinemia	2 (1.4)
	Langerhans cell histiocytosis	1 (0.7)
Cancer treatments	Chemotherapy	83 (57.6)
	Bone Marrow Transplant	35 (24.3)
	Completed treatment	17 (11.8)
	No treatment	9 (6.3)
Febrile	Yes	91 (63.2)
	No	53 (36.8)
Patients who died	Yes	12 (8.3)
	No	132 (91.7)

*ALL* acute lymphoid leukemia, *AML* *acute myeloid leukemia*, *MM* multiple myeloma, *CLL* chronic lymphoid leukemia

### Microbial profiles and site of isolation

A total of 144 microbial samples were collected, the majority isolated from blood (57, 39.6%), urine (40, 27.8%) and sputum (17, 11.8%). A smaller portion was isolated from fluid (3, 2.1%; one from ascetic fluid and the other two unspecified), stool (2, 1.4%) and cerebrospinal fluid (1, 0.7%).

There were 144 positive cultures; 66 (45.8%) isolates were GNB, 57 (39.6%) GPB, and fungal infections were positive in 21 (14.6%) samples.

Among GNB, *Pseudomonas aeruginosa* (27, 40.9%) was predominant, followed by *E. coli* (20, 30.3%) which can be divided into non-extended-spectrum beta-lactamase-producing *Escherichia coli* (non-ESBL-EC); (10, 50%), and ESBL-producing *Escherichia coli* (ESBL-EC); (10, 50%). These were followed by both *Acinetobacter baumannii* and *Klebsiella pneumonia* (6, 9.1%) (Table 2).

**Table 2:** Frequency and percentage of isolated microorganisms

Microorganism	Frequency
Total number 144	N (%)
Gram-negative, total	**66 (45.8)**
*Pseudomonas aeruginosa*	27 (40.9)
*E. coli*	20 (30.3)
Non*-*ESBL	10 (50)
ESBL	10 (50)
*Acinetobacter baumannii*	6 (9.1)
*Klebsiella pneumonia*	6 (9.1)
*Enterobacter cloacae*	2 (3.2)
*Salmonella* species	1 (1.5)
*Sphingomonas paucimobilis*	1 (1.5)
Shigella species	1 (1.5)
*Raoultella planticola*	1 (1.5)
*Haemophilus influenzae*	1 (1.5)
Gram-positive, total	57 (39.6)
*Staphylococcus epidermidis*	14 (24.6)
*Enterococcus faecium*	10 (17.5)
*Staphylococcus haemolyticus*	10 (17.5)
*Staphylococcus hominis*	6 (10.5)
*Enterococcus faecalis*	5 (8.7)
*Staphylococcus aureus*	4 (7.0)
*Streptococcus agalactia*	2 (3.5)
*Micrococcus luteus*	2 (3.5)
*Streptococcus oralis*	1 (1.8)
*Staphylococcus capitis*	1 (1.8)
*Staphylococcus sciuri*	1 (1.8)
*Kocuria varians*	1 (1.8)
Fungal infection	**21 (14.6)**
Candida species	**20 (95.2)**
*Candida tropicalis*	8 (38.1)
*Candida glabrata*	7 (33.3)
*Candida albicans*	4 (19)
*Candida dubliniensis*	1 (4.8)
*Saprochaete capitate*	**1 (4.8)**

*E. coli* *Escherichia coli*, *ESBL* extended-spectrum beta-lactamase

In terms of GPB, *Staphylococcus epidermidis* represented the most frequent species (14, 24.6%), followed by *Enterococcus faecium* and *Staphylococcus hemolyticus* (both 10, 17.5%), *Staphylococcus hominis* (6, 10.5%), *Enterococcus faecalis* (5, 8.7%), and *Staphylococcus aureus* (4, 7.0%) (Table 2).

Fungal infections were positive in 21 samples (14.6%). The vast majority were *Candida* spp. (20, 95.2%) with only one (4.8%) *Saprochaete capitate* (Table 2). Among the 20 isolated *Candida* spp., eight were *Candida tropicalis,* seven were *Candida glabrata*, four were *Candida albicans,* and one was *Candida dubliniensis.*

### Antimicrobials used before and after culture

The vast majority of antimicrobials were used in combinations. Among those used in combination, amikacin was the most popular antibiotic for empiric therapy, since it was used in 45 cases out of 144 organisms (31.3%, 45/144), followed by vancomycin (30.5%, 44/144), meropenem (27.8%, 40/144), piperacillin-tazobactam (27.8%, 40/144), colistin (23.6%, 34/144) and tigecycline (18.6%, 27/144). On the other hand, the least frequently used antimicrobials were azithromycin, clindamycin, and caspofungin (0.7, 1/144) (Table 3).

**Table 3:** Frequency and percentage of empiric antimicrobials (before the results of the culture)

Empiric antimicrobials	Frequency N (%)
Amikacin*	45 (31.3)
Vancomycin*	44 (30.3)
Meropenem*	40 (27.8)
Meropenem**	2 (1.4)
Piperacillin-Tazobactam*	40 (27.8)
Piperacillin- Tazobactam**	1 (0.7)
Colistin*	34 (23.6)
Colistin**	1 (0.7)
Tigecycline*	27 (18.6)
Voriconazole*	21 (14.6)
Fluconazole*	18 (12.5)
Miconazole*	18 (12.5)
Levofloxacin*	16 (11.1)
Levofloxacin**	1 (0.7)
Trimethoprim-Sulfamethoxazole*	15 (10.4)
Ceftazidime*	10 (6.9)
Acyclovir*	10 (6.9)
Metronidazole*	8 (5.6)
Metronidazole**	1 (0.7)
Ciprofloxacin*	6 (4.5)
Ciprofloxacin**	1 (0.7)
Ceftriaxone*	4 (2.8)
Azithromycin*	1 (0.7)
Azithromycin**	1 (0.7)
Amphotericin*	2 (1.4)
Caspofungin*	1 (0.7)
Clindamycin*	1 (0.7)

^*^Used in combination therapy, **used as monotherapy

For culture-guided antibiotic use, the antibiotic most frequently used was meropenem (41.7%, 60/144), followed by colistin (33.3%, 48/144), tigecycline (26.4%, 38/144), vancomycin (25%, 36/144) and amikacin (22.9%, 33/144). Meanwhile, the antibiotics used most frequently were linezolid (0.7%, 1/144), followed by amoxicillin-clavulanate (1.4%, 2/144) (Table 4). De-escalation of therapy occurred in 52 (36.1%) cases in the sample.

**Table 4:** Frequency and percentage of antimicrobials after the results of the culture

Antimicrobials	Frequency N (%)
Meropenem	60 (41.7)
Colistin	48 (33.3)
Tigecycline	38 (26.4)
Vancomycin	36 (25)
Amikacin	33 (22.9)
Fluconazole	21 (14.6)
Voriconazole	26 (18.1)
Miconazole	23 (16)
Piperacillin-Tazobactam	20 (13.9)
Levofloxacin	16 (11.1)
Acyclovir	15 (10.4)
Trimethoprim-Sulfamethoxazole	14 (9.7)
Metronidazole	8 (5.6)
Ciprofloxacin	8 (5.6)
Ceftazidime	7 (4.9)
Ceftriaxone	5 (3.5)
Amphotericin B	5 (3.5)
Caspofungin	4 (2.8)
Clindamycin	3 (2.1)
Amoxicillin-Clavulanate	2 (1.4)
Linezolid	1 (0.7)

### Antimicrobial resistance of gram-negative bacterial isolates

In general, the highest resistance rates for GNB were 79.3% for ampicillin, 73.3% to levofloxacin, 65.7% for ceftriaxone, 61.8% to piperacillin, and 59.3%, 56.5% and 59.4% for cefuroxime, ciprofloxacin, and tobramycin, respectively. Meanwhile, the lowest resistance rates were 3.6% for ertapenem**,** 6.25% for nitrofurantoin, 8.3% to colistin, and 16.3% to piperacillin-tazobactam. Among GNB, 23 isolates (34.8%) were ESBL and 23 isolates (31.8%) were CRE (carbapenem resistant *Enterobacteriaceae*). The most common isolated GNB, *Pseudomonas aeruginosa,* had a high resistance rate to ciprofloxacin (60%), imipenem (59.3%), piperacillin (54.2%), meropenem, and gentamicin (48% each). Furthermore, resistance rates against cephalosporins, cefepime, and ceftazidime were 16% and 24% respectively. *E. coli* isolates were highly susceptible to amikacin (95%), while they were highly resistant to trimethoprim-sulfamethoxazole and fluoroquinolones. Regarding the six isolates of *Acinetobacter baumannii,* the highest resistance rates were towards carbapenems (80% for meropenem and 83.3% to imipenem), piperacillin-tazobactam (83.3%) and gentamicin (66.7%). Only four isolates were tested for sensitivity to colistin and all showed 0% resistance. Finally, the six isolates of *Klebsiella pneumonia* were highly susceptible to piperacillin-tazobactam, carbapenems, fluoroquinolones ceftazidime and cefepime*.* The antimicrobial resistance profiles of the most frequently isolated GNB are reported in Table 5.

**Table 5:** Susceptibility of the most common gram-negative bacterial isolates tested

Antibiotics	Ampicillin	Amoxicillin-Clavulanic acid	Piperacillin	Piperacillin-Tazobactam	Imipenem	Meropenem	Ertapenem	Cefotaxime	Cefepime	Cefuroxime
*Susceptibility (%)*										
GNB (total = 66)	20.7	53.4	38.2	83.7	64.6	71.1	96.4	61.3	68.3	40.7
*pseudomonas aeruginosa* (27)	44.4	8.3	45.8	93.75	40.7	52	–*	33.3	84	0
*E. coli* (20) (ESBL, 10; NON-ESBL, 10)	22.2	77.8	0*	87.5	90	95	94.4	58.8	50	58.3
*Acinetobacter baumannii* (6)	–	0	0	16.7	16.7	20	–	0	16.7	25
*Klebsiella pneumonia* (6)	0	100	–	100	100	100	100	100	100	100

Antibiotics	Ceftriaxone	Ceftazidime	Ciprofloxacin	Levofloxacin	Trimethoprim-Sulfamethoxazole	Gentamicin	Tobramycin	Amikacin	Nitrofurantoin	Colistin
*Susceptibility (%)*										
GNB (total = 66)	34.3	64.5	43.5	26.7	58.3	61.7	40.6	71.2	93.75	91.7
*pseudomonas aeruginosa* (27)	7.7	76	40	21.4	100*	52	42.3	44.4	–	93.3
*E. coli* (20) (ESBL, 10; NON-ESBL, 10)	46.2	50	30	25	36.8	70	–	95	92.9	66.7**
*Acinetobacter baumannii* (6)	0	16.7	16.7	0	100	33.3	25	100*	–	100
*Klebsiella pneumonia* (6)	100	100	100	100*	100	100	–	100	100	–

*GNB* Gram negative bacteria, *E. coli* *Escherichia coli*,* ESBL* extended-spectrum beta-lactamase

–, not tested

*Only one bacterial isolate was tested

**Only two bacterial isolates were tested

### Antimicrobial resistance of gram-positive bacterial isolates

Among the CoNS isolated in our study (*S. epidermidis, hemolyticus, hominis, sciuri,* and *capitis*( and *Staphylococcus aureus,* none were resistant to vancomycin or linezolid, while 93.3% of CoNS and 75% of *Staphylococcus aureus* were resistant to oxacillin. Regarding the 14 isolates of *Staphylococcus epidermidis* isolates, all were resistant to penicillin and cephalosporins and 54.5% were resistant to trimethoprim-sulfamethoxazole. Among the 10 isolates of *Enterococcus faecium* and 5 of *Enterococcus faecalis*, 90% of *Enterococcus faecium* isolates were resistant to vancomycin (VRE) while none of the *Enterococcus faecalis* isolates were VRE. 40% of *Enterococcus faecium* isolates were resistant to streptomycin, 30% were resistant to gentamicin, and 11.1% were resistant to tigecycline. Meanwhile, *Enterococcus faecalis* species had 80% resistance to streptomycin, 50% resistance to gentamicin, and 33.3% resistance to tigecycline. However, none of the *Enterococcus faecalis* or *Enterococcus faecium* isolates were resistant to linezolid. Overall, the highest resistance rates of GPB were 91.1% to oxacillin, 88.8% to amikacin, 86.9% to cefuroxime, 85.1% to erythromycin, and 84.8%, 82.6%, 77.4 to penicillin, ceftriaxone, amoxicillin-clavulanic, respectively. Meanwhile, the lowest resistance rates were 0% to linezolid, 4.2% to tigecycline, 11.5% to quinupristin-dalfopristin, and 16.1% to vancomycin. The antimicrobial resistance profiles of the most frequently isolated GPB are reported in Table 6.

**Table 6:** Susceptibility of the most common gram-positive bacterial isolates tested

Antibiotics	Ampicillin	Amoxicillin-Clavulanic acid	Penicillin	Oxacillin	Cefuroxime	Ceftriaxone	Clindamycin	Erythromycin	Quinupristin/ dalfopristin	Ciprofloxacin	Moxifloxacin
*Susceptibility (%)*											
GPB (total = 57)	55	22.6	15.2	8.9	13.1	17.4	28.9	14.9	88.5	37.1	65.9
CoNS (32)	–	0	6.9	6.7	7.2	6.2	20	6.7	96.8	35.5	58.1
*Staphylococcus epidermidis* (14)	–	0	0	0	0	0	7.2	0	100	23.1	46.2
*Staphylococcus haemolyticus* (10)	–	0	0	0	0	0	20	0	100	10	40
*Staphylococcus hominis* (6)	–	0	16.7	0	0	0**	33.3	0	83.3	83.4	100
*Staphylococcus sciuri* *(1)*	–	100	–	100	100	–	–	100	100	100	100
*Staphylococcus capitis (1)*	–	100	100	100	–	100	100	100	100	100	100
*Enterococcus faecium* (10)	100	0	0	–	0	100*	–	0	100	10	50**
*Enterococcus faecalis* (5)	20	80	100	100	0	0	100	0	0	20	100
*Staphylococcus aureus* (4)	0*	25	0	25	33.4	50**	75	50	100	75	100

Antibiotics	Levofloxacin	Trimethoprim-Sulfamethoxazole	Gentamicin	Streptomycin	Amikacin	Vancomycin	Tetracycline	Tigecycline	Rifampicin	Linezolid	
*Susceptibility (%)*											
GPB (total = 57)	42.9	60	62	50	11.2	83.9	58.8	95.8	71.9	100	
CoNS (32)	38.8	55.2	55	–	0	100	71.5	100	67.9	100	
*Staphylococcus epidermidis* (14)	38.5	45.5	53.8	–	0	100	63.6	100	100	100	
*Staphylococcus haemolyticus* (10)	0	55.6	20	–	0	100	66.7	100	80	100	
*Staphylococcus hominis* (6)	83.3	40	100	–	–	100	83.3	100	20	100	
*Staphylococcus sciuri* *(1)*	100	100	100	–	–	100	100	100	100	100	
*Staphylococcus capitis (1)*	100	100	100	–	–	100	100	100	100	100	
*Enterococcus faecium* (10)	10	–	70	60	0	10	50	88.9	–	100	
*Enterococcus faecalis* (5)	20	100	50	20	0	100	0	66.7	–	100	
*Staphylococcus aureus* (4)	100	100	100	100*	100	100	50	75	100	100	

*GPB* Gram Positive Bacteria, *CoNS* coagulase negative staphylococci

–, not tested

*Only one bacterial isolate was tested

**Only two bacterial isolates were tested

### Antifungal resistance and sensitive profiles of fungal organisms

As for antifungals, no resistance was found to caspofungin, fluconazole, flucytosine, voriconazole, or micafungin.

### Multi-drug resistant organisms (MDRO)

MDROs are defined as those resistant to at least one agent in three or more antimicrobial categories [24]. 51.5% of GNB isolates and 68.4% of GPB isolates were found to be MDRO. Among GNB, *Acinetobacter baumannii* had the highest rate of MDRO (83.3%), whereas among GPB, CoNS had the highest rate (81.3%) (Table 7).

**Table 7:** Frequency and percentage of multidrug resistant organisms

Microorganism	MDRO n (%)	DTR n (%)
GNB (66)	**34 (51.5)**	**11 (16.7)**
*Pseudomonas aeruginosa* (27)	15 (55.6)	5 (18.5)
*E. coli* (20)	13 (65)	1 (5)
*Acinetobacter baumanni* (6)	5 (83.3)	5 (83.3)
*Klebsiella pneumonia* (6)	0 (0)	0 (0)
*Enterobacter* spp. (5)	1 (20)	0 (0)
GPB (57)	**39 (68.4)**	**–**
CoNS (32)	26 (81.3)	**–**
*Enterococcus* spp. (15)	11 (73.3)	**–**
*Staphylococcus aureus* (4)	2 (50)	**–**

*Enterobacter* spp.: *Enterobacter cloacae, Salmonella* species*, Raoultella planticola,* and *Shigella* species; *Enterococcus* spp.: *Enterococcus faecium* and *Enterococcus faecalis*

*MDRO* multidrug resistant organisms, *GNB* Gram-Negative Bacteria, *GPB* Gram-Positive Bacteria, *E. coli* *Escherichia coli,* *spp.* Species, *CoNS* coagulase-negative Staphylococci, *DTR* difficult-to-treat resistance

### Difficult-to-treat resistance (DTR) of GNB

DTR is defined as an isolate demonstrating intermediate or resistant phenotype to all reported agents in carbapenem, β-lactam, and fluoroquinolone categories, including additional agents, such as piperacillin-tazobactam and ampicillin-sulbactam (*A. baumannii* only), and aztreonam (not applicable to *A. baumannii*), when results are available [25]. In this study, 11 isolates (16.7%) were DTR; 5 (18.5%) were *Pseudomonas aeruginosa*, 5 (83.3%) were *Acinetobacter baumanni* (6) and 1 (5%) was *E. coli* (Table 7).

## Discussion

Infections are the most common cause of death in cancer patients, especially among those with hematologic malignancies, with studies reporting that approximately 60% of deaths are infection-related [26]. This increased risk of infections can be due to host or treatment-related causes. Host-related factors consist of immunodeficiency, comorbid illnesses, mucosal ulcerations, previous infections, nutritional deficiency, and stress [26], while treatment-related factors include invasive procedures, surgery, radiation, immunosuppressive drugs, and use of antimicrobials [27]. These infections can be caused by various pathogens such as viruses, bacteria, fungi, etc. Bacteria are the leading cause of infections in cancer patients, followed by fungi [27].

In our study, *Pseudomonas aeruginosa* (27, 43.6%) was the predominant bacterium among GNB, followed by *E. coli* (20, 32.3%) that can be divided into non-ESBL (10, 50%) and ESBL-*E. coli* (10, 50%). These were followed by *Acinetobacter baumannii* and *Klebsiella pneumonia*, with six isolates each (9.7%). These results are in conjunction with other studies conducted in India and Pakistan. In the former, they reconfirmed the predominance of GNB in patients with hematologic cancers, with *E. coli, Pseudomonas,* and *Klebsiella* having the largest shares [28] In the latter study, which evaluated GNB isolated from bloodstream infections of patients on chemotherapy, *Pseudomonas aeruginosa* was the most frequent bacteria, followed *by E. coli*, *Klebsiella*, *Proteus*, and *Shigella* [29]. These results are also similar to a study conducted in Italy, where *E. coli* was the most frequent organism, followed by *Pseudomonas aeruginosa*, *Klebsiella pneumoniae*, and *Enterobacter cloacae* [30]. In another study carried out in Sudan, *E. coli* represented the most frequently isolated bacterium among GNB, followed by *Pseudomonas aeruginosa* [31]. Meanwhile, a study conducted in Egypt found that the GNB most frequently isolated from all samples was *Klebsiella pneumoniae* followed by *E. coli* [32].

Regarding GPB, CoNS represented the most frequent species isolated in our study (32, 56.1%), followed by *Enterococcus faecium* (10, 17.5%), *Enterococcus faecalis* (5, 8.7%) and *Staphylococcus aureus* (4, 7.0%). These results are comparable to the aforementioned Italian study, where CoNS were the most common species, followed by *Enterococcus* spp., viridans group streptococci (VGS) and *Staphylococcus aureus* (11). In the Indian study, the most frequent GPB isolates were CoNS, then *Staphylococcus aureus*, *Streptococcus* spp., and *Enterococcus* spp. (14).

In our study, the bacteria most commonly isolated were *Pseudomonas aeruginosa* (22%), *E. coli* (16.3%), and *Staphylococcus epidermidis* (11.4%), followed by *Enterococcus faecium* and *Staphylococcus haemolyticus* (8.1% each), and then *Klebsiella pneumonia, Acinetobacter baumanii,* and *Staphylococcus hominis* (4.9% each). In comparison, when looking at patients with hematologic malignancies in Japan, *E. coli* was the most commonly seen bacterium, followed by *Klebsiella* spp., *Pseudomonas aeruginosa*, *Staphylococcus aureus*, *Enterobacter* spp. *Citrobacter* spp., and *Acinetobacter* spp. [33].

Hard to spot but lethal if missed, invasive fungal infections—predominantly caused by *Aspergillus* and *Candida—*are the leading infectious cause of mortality in patients with myelosuppression due to chemotherapy [34]. In our study, *Candida* had the highest share of fungal infections, in contrast to a study in Italy where most infections were caused by *Aspergillus* spp., followed by *Candida* [35].

In our study, *Pseudomonas aeruginosa* exhibited high resistance to ciprofloxacin (60%), in concordance with numbers found in similar Italian studies [30, 36], and with a Spanish study that observed resistance to ciprofloxacin among cancer patients in general [37]. *Pseudomonas aeruginosa* isolates in our study also had high resistance to carbapenems, including imipenem (59.3%), meropenem (48%), and gentamicin (48%). These numbers resemble those found in another study where the resistance rate to carbapenems was 60% [36], and in an Italian study where the resistance rate to meropenem was 71.2% [30]. However, this is in contrast to an American study that found the resistance to imipenem seen among solid and hematological cancer patients was only 6% [38]. Also among our *Pseudomonas aeruginosa* isolates, piperacillin resistance was found to be 54.2%, while in a previously mentioned study it was found to be 24% [36]. Meanwhile, among cephalosporins, cefepime, and ceftazidime, resistance rates were 16% and 24% respectively. The reasons behind these low rates of resistance to cephalosporins are the infrequent use of these agents, as the prescription of cefepime is highly restricted, and piperacillin-tazobactam is the most commonly used initial therapy for neutropenic fever instead. This highlights the importance of diversification of antibiotic use, such as prescribing third-generation cephalosporins (ceftazidime) for neutropenic fever [39], to avoid selection of carbapenem resistance by extensive carbapenem use. However, the selection of empiric antimicrobial therapy should be based on multiple factors, including but not limited to the clinical status of the patient, previous cultures and colonization, and institutional antibiograms [40, 41].

Among GNB, 21 CRE (31.8%) were detected, more than that seen in febrile neutropenic patients with hematological cancer in Japan [42]. In our study, the resistance of *E. coli* isolates to amikacin was only 5%, similar to the results of another study where 85.2% of *E. coli* isolates were found to be sensitive to amikacin [30]. On the other hand, ESBL-*E. coli* exhibited 100% resistance to both cephalosporins and ampicillin, similar to previous research, where the vast majority of ESBL-producing isolates were resistant to all generations of cephalosporins [42]. *E. coli* in our study also exhibited high resistance to levofloxacin and TMP-SMX (75% and 63.2%, respectively), similar to the results found in a previous study [30]. This could be due to the frequent use of fluoroquinolones (especially levofloxacin) for prophylaxis in patients with prolonged neutropenia [39].

Regarding the six isolates of *Acinetobacter baumannii*, the highest resistance rates were observed to carbapenems (80% to meropenem and 83.3% to imipenem) and piperacillin-tazobactam (83.3%) similar to a related study held in Turkey [43]. Isolates also exhibited high resistance to gentamicin (66.7%). Four of these isolates were tested for resistance to colistin and all were sensitive, in agreement with prior research where all isolates of *Acinetobacter baumannii* were susceptible to colistin [44]. Finally, the six isolates of *Klebsiella pneumonia* were 100% susceptible to piperacillin/tazobactam, carbapenems, fluoroquinolones, ceftazidime, and cefepime. In other studies, 55.8% of *Klebsiella* isolates were resistant to piperacillin/tazobactam [30], 44.9% were resistant to meropenem while 1% were resistant to imipenem [38], 69.8% were resistant to ciprofloxacin, 58.1% were resistant to ceftazidime [30], and 20% were resistant to cefepime [42].

Among the 10 *Enterococcus faecium isolates* and the 5 *Enterococcus faecalis* isolates*,* 90% of *Enterococcus faecium* isolates were VRE while none of the *Enterococcus faecalis* isolates were VRE. Regarding *Enterococcus faecium*, 40% of isolates were resistant to streptomycin, 30% were resistant to gentamicin, and 11.1% were resistant to tigecycline. Meanwhile, *Enterococcus faecalis* species had 80% resistance to streptomycin, 50% resistance to gentamicin, and 33.3% resistance to tigecycline. In particular, none of the *Enterococcus faecalis* or *Enterococcus faecium* isolates was resistant to linezolid, in agreement with prior research [30].

Among the CoNS (*Staph. epidermidis, hominis* and *haemolyticus)*, no isolates were resistant to vancomycin or linezolid, while 93.3% were resistant to oxacillin, similar to the results of a previous study [30]. Regarding the 14 isolates of *Staphylococcus epidermidis*, all were resistant to penicillin and cephalosporins, and 54.5% were resistant to trimethoprim-sulfamethoxazole. Regarding the four isolates of *Staphylococcus aureus*, 75% were resistant to oxacillin, a high percentage compared to patients in Italy (36.4%) [30]. Additionally, 66.6% were resistant to cefuroxime and 50% were resistant to ceftriaxone. However, all were sensitive to both vancomycin and linezolid, similar to those in the former Italian study [30].

Regarding antifungal resistance rates, all were sensitive to caspofungin, comparable to a similar study in which caspofungin resistance rates were 5% [45]. All were sensitive to fluconazole, voriconazole, flucytosine, and micafungin. When reviewing the literature on *Candida* infections in patients with hematologic malignancies, a study showed that 27.6% [37] were resistant to fluconazole. Meanwhile, in another study, 8% of *Candida* were resistant to voriconazole and 5% were resistant to caspofungin [45].

51.5% of GNB and 68.4% of GPB in this study were MDRO. Among GNB, *Acinetobacter baumanni* had the highest rate of MDRO (83.3%), whereas among GPB, CoNS had the highest rate (81.3%). Meanwhile, in a similar study in which MDROs were isolated in 13% of patients, the most frequently isolated MDRO was *Klebsiella pneumoniae*, followed by MRSA, *Acinetobacter baumanni*, *Pseudomonas*, *E. coli,* and VRE [46].

This study is the first in Palestine to determine the microbial profile of infections in patients with hematological malignancies. However, there were some limitations to our study. First, not all data were written in the patient’s medical reports such as white blood cell counts, absolute neutrophil counts, and patient temperatures at the time of culture, so we could not assess neutropenic fever and its relationship with other variables. Furthermore, some data were not collected, such as the last time a patient received a chemotherapy session. Second, our data were collected from only one center that may not be representative of other centers. Third, some patients died or left the hospital before the culture results were ready, so they did not receive any treatment other than empirical antibiotics. Finally, the study did not assess increases in antibiotic resistance year over year.

## Conclusions

Patients with hematologic malignancies are at risk for a variety of serious infections that cause significant morbidity and mortality. The most common bacterial isolates among GNB were *Pseudomonas aeruginosa* and *E. coli,* while coagulase-negative staphylococci and *Enterococcus faecium* were the most common among GPB. Our study showed alarming rates of resistance to the most widely used antibiotics, thus highlighting the need to develop local guidelines for antimicrobial use based on local resistance patterns of these organisms. This study also emphasizes the need to develop antimicrobial stewardship programs in local hospitals. Enforcing the implementation of infection control policies would help curb the spread of these MDROs and reduce the morbidity, mortality, and economic burden of these serious infections.

## Data Availability

The data sets used and/or analysed during this study are available from the corresponding author on reasonable request.

## Abbreviations

GNB: Gram-negative bacteria
GPB: Gram-positive bacteria
ALL: Acute lymphoid leukemia
AML: Acute myeloid leukemia
MM: Multiple myeloma
CLL: Chronic lymphoid leukemia
*E. coli*: *Escherichia coli*
ESBL: Extended-spectrum beta-lactamase
IRB: Institutional Review Board
SD: Standard deviation
CoNS: Coagulase negative staphylococcus
VRE: Vancomycin resistance enterococcus
MDRO: Multidrug resistant organisms
spp.: Species
CRE: Carbapenem-resistant *Enterobacteriaceae*
DTR: Difficult-to-treat resistance
